# Ubiquitination and sumoylation of the HTLV-2 Tax-2B protein regulate its NF-κB activity: a comparative study with the HTLV-1 Tax-1 protein

**DOI:** 10.1186/1742-4690-9-102

**Published:** 2012-12-07

**Authors:** Marco Turci, Julie Lodewick, Gianfranco Di Gennaro, Anne Sophie Rinaldi, Oriano Marin, Erica Diani, Carla Sampaio, Françoise Bex, Umberto Bertazzoni, Maria Grazia Romanelli

**Affiliations:** 1 Department of Life and Reproduction Sciences, Section of Biology and Genetics, University of Verona, Strada Le Grazie 8, 37134, Verona, Italy; 2 Institute for Microbiological Research J-M Wiame, Laboratory of Microbiology, Université Libre de Bruxelles, 1, Avenue E. Gryson, Brussels, Belgium; 3 Department of Oncology and Surgical Sciences, University of Padova, Padova, Italy

**Keywords:** HTLV-1, HTLV-2, Retrovirus, Tax, Oncoprotein, Leukemia, Post-translational modification, Ubiquitination, Sumoylation, NF-κB pathway

## Abstract

**Background:**

Retroviruses HTLV-1 and HTLV-2 have homologous genomic structures but differ significantly in pathogenicity. HTLV-1 is associated with Adult T cell Leukemia (ATL), whereas infection by HTLV-2 has no association with neoplasia. Transformation of T lymphocytes by HTLV-1 is linked to the capacity of its oncoprotein Tax-1 to alter cell survival and cell cycle control mechanisms. Among these functions, Tax-1-mediated activation of cellular gene expression via the NF-κB pathway depends on Tax-1 post-translational modifications by ubiquitination and sumoylation. The Tax-2 protein of HTLV-2B (Tax-2B) is also modified by ubiquitination and sumoylation and activates the NF-κB pathway to a level similar to that of Tax-1. The present study aims to understand whether ubiquitination and sumoylation modifications are involved in Tax-2B-mediated activation of the NF-κB pathway.

**Results:**

The comparison of Tax-1 and Tax-2B lysine to arginine substitution mutants revealed conserved patterns and levels of ubiquitination with notable difference in the lysine usage for sumoylation. Neither Tax-1 nor Tax-2B ubiquitination and sumoylation deficient mutants could activate the NF-κB pathway and fusion of ubiquitin or SUMO-1 to the C-terminus of the ubiquitination and sumoylation deficient Tax-2B mutant strikingly restored transcriptional activity. In addition, ubiquitinated forms of Tax-2B colocalized with RelA and IKKγ in prominent cytoplasmic structures associated with the Golgi apparatus, whereas colocalization of Tax-2B with the RelA subunit of NF-κB and the transcriptional coactivator p300 in punctate nuclear structures was dependent on Tax-2B sumoylation, as previously observed for Tax-1.

**Conclusions:**

Both Tax-1 and Tax-2 activate the NF-κB pathway via similar mechanisms involving ubiquitination and sumoylation. Therefore, the different transforming potential of HTLV-1 and HTLV-2 is unlikely to be related to different modes of activation of the canonical NF-κB pathway.

## Background

Human T-cell leukemia viruses type 1 (HTLV-1) and type 2 (HTLV-2) share a common genomic structure but differ significantly in their pathogenic properties [1,2]. This difference is generally attributed to the properties of their transactivating Tax proteins, Tax-1 and Tax-2, both of which activate gene expression via ATF/CREB and NF-κB pathways [3]. The transforming activity of Tax-1 is linked to its ability to activate the NF-κB pathway, but also to promote cell cycle progression, genome instability and inactivation of the p53 and pRb tumor suppressors resulting in the survival and proliferation of HTLV-1 infected T-cells [4-11].

Because less is known about Tax-2, a comparative analysis between Tax-1 and Tax-2 is important in order to reach a better understanding of the differences in pathogenesis. In a recent review [12], the known features and functional differences of Tax-1 and Tax-2 were discussed. Although Tax-1 and Tax-2B share 85 percent of amino acid similarity, two basic structural features differentiate the two proteins. First, a domain outlined by amino acids 225 and 232 of Tax-1 is responsible for p100 processing into p52 leading to activation of the non-canonical NF-κB pathway [13,14]. Second, the C-terminus of Tax-1 contains a domain involved in micronuclei formation [15] and a PDZ binding motif (PBM) encompassing the four C-terminal amino acids responsible for the binding to several PDZ domain-containing proteins [16-18]. In addition, some HTLV-2 subtypes express shorter versions of Tax-2 (namely Tax-2A and Tax-2CG) which, contrary to Tax-2B, do not functionally inactivate p53 [10,19].

Recent studies have demonstrated a hierarchical sequence of post-translational modifications that control Tax-1 intracellular localization and transcriptional activities (reviewed in [20,21]). Phosphorylation-dependent ubiquitination controls both Tax-1 retention in the cytoplasm and subsequent targeting to Golgi-associated structures, in which Tax-1 colocalizes with RelA and IKKγ leading to activation of the IκB kinase (IKK) complexes [22-25]. These steps determine phosphorylation and degradation of the NF-κB inhibitor IκBα and the migration of the active RelA subunit of NF-κB to the nucleus. In the nucleus, Tax-1 is polysumoylated at lysine residues K7 and K8 at amino acid positions 280 and 284. Polysumoylation determines the retention of Tax-1 in the nucleus, the formation of Tax nuclear bodies (NBs) and the recruitment within these NBs of various cellular transcription factors, including the RelA subunit of NF-κB [26] and the transcriptional coactivator p300 [27]. Thus, phosphorylated and polyubiquitinated Tax-1 molecules in the cytoplasm, as well as polysumoylated Tax-1 molecules in the nucleus, cooperate for activation of the NF-κB pathway [22,25,28].

The recent findings that Tax-2B is also modified by ubiquitination and sumoylation [29] and that Tax-1 and Tax-2B form complexes and colocalize with the same cellular factors [30] point to a common intracellular distribution and interactome. To determine whether ubiquitination and sumoylation control Tax-2B transcriptional activity and to outline possible differences between Tax-1 and Tax-2B, we have constructed a series of Tax-2B mutants with substitution of specific lysine residues by arginines as well as C-terminal fusions of these mutants to ubiquitin or SUMO-1. We have compared the ubiquitination and sumoylation status of the Tax-2B mutants, their intracellular distribution and their capacity to activate gene expression via the NF-κB pathway, with the patterns of the corresponding Tax-1 mutants. This study reveals that the transcriptional activity of the Tax-2B lysine to arginine substitution mutants and the ubiquitin and SUMO-1 fusions correlate with their ubiquitination and sumoylation status, suggesting a common mechanism of NF-κB activation for Tax-1 and Tax-2B.

## Results

### Mutation of multiple lysine residues determines loss of ubiquitination and sumoylation of both Tax-1 and Tax-2B

**Figure 1:**
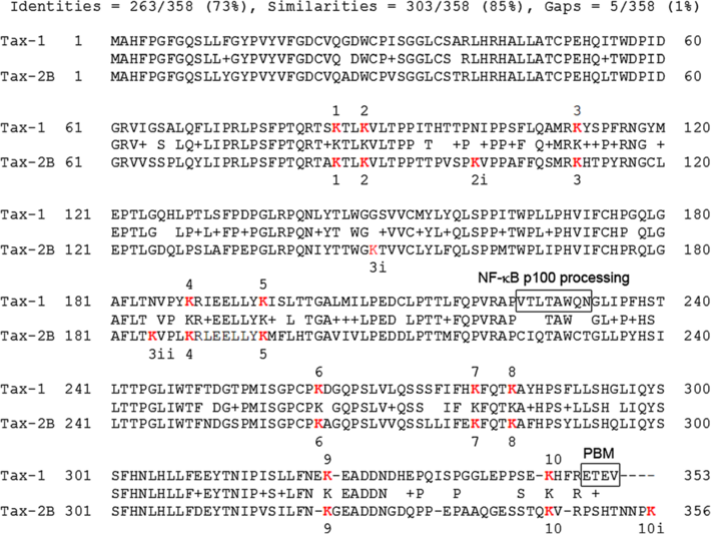
**Alignment of the Tax-1 and Tax-2B amino acid sequences. **The 10 lysine residues of Tax-1 and the 10 equivalent lysine residues of Tax-2B are designated K1 to K10. The four additional Tax-2B lysine residues are designated K2i, K3i, K3ii and K10i. The two functional domains involved in Tax-1-mediated processing of NF-κB p100 and binding to PDZ domain containing protein (PBM) that are divergent in Tax-2B, are highlighted.

Previous studies indicated that Tax-1 is polysumoylated at lysine residues K7 and K8 and polyubiquitinated at the five central lysine residues K4 to K8 [22]. These five amino acids are maintained with similar sequence environment in the Tax-2B amino acid sequence. The comparative alignment of the Tax-1 and Tax-2B amino acid sequences is shown in Figure 1. The nomenclature of the 14 lysine residues in Tax-2B was designed to maintain identical numbers for equivalent lysine residues in the two sequences.

**Figure 2:**
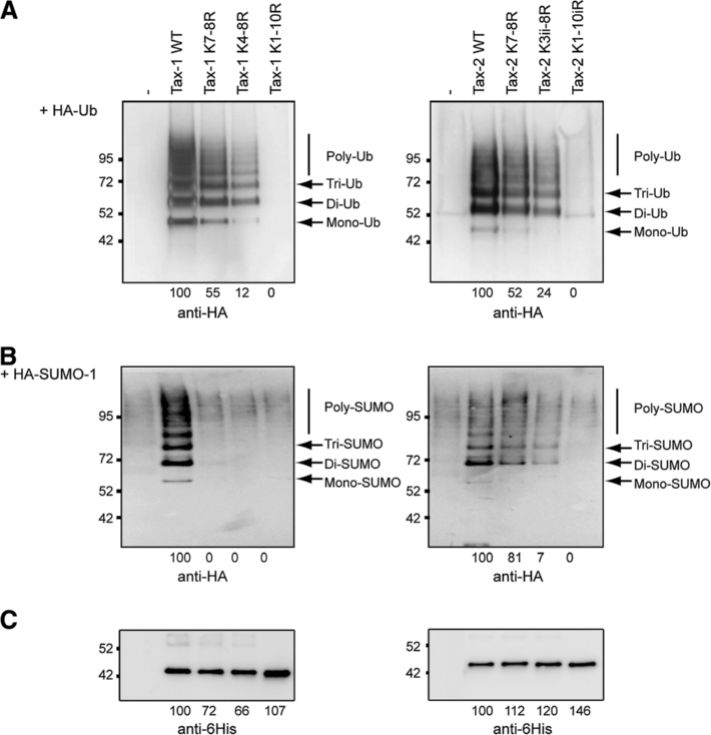
**Mutation of multiple Tax-1 or Tax-2B lysine residues leads to ubiquitination and sumoylation deficiency. **293T cells were cotransfected with vectors expressing wild type (WT) or lysine to arginine substitution mutants of Tax-1-6His and Tax-2B-6His, and with vectors expressing (**A**) HA-Ubiquitin or (**B**) HA-SUMO-1. Purification of the ubiquitinated and sumoylated forms was performed by Ni-NTA pulldown and the purified proteins were analyzed by Western Blot using an anti-HA antibody. (**C**) Comparable levels of wild type and mutant Tax proteins in the Ni-NTA pulldown preparations were ascertained by Western blot analysis using an anti-6His antibody. The abundance of the different species on the blots was estimated, and the percentages relatively to wild type species are indicated under each Western blot image.

To investigate the role of post-translational modifications of Tax-2B in activating the NF-κB pathway, we first constructed Tax-2B lysine to arginine substitution mutants equivalent to Tax-1 mutants K7-8R (sumoylation deficient) as well as K4-8R and K1-10R (sumoylation and ubiquitination deficient) mutants, as described in [22]. The generated mutants Tax-2 K7-8R, K3ii-8R and K1-10iR were fused to a C-terminal 6 histidine tag. Mutants were coexpressed with either HA-Ubiquitin or HA-SUMO-1 and purified by Ni-NTA pulldown. The extent of ubiquitination and sumoylation in comparison with the equivalent Tax-1 mutants is shown in Figure 2.

The ladders of ubiquitinated forms were similar for wild type Tax-1 and Tax-2B, though a lower rate of mono-ubiquitination was observed for Tax-2B (Figure 2A). The abundance of the ubiquitinated forms was progressively reduced with increasing number of substituted lysine residues, ultimately leading to undetectable ubiquitinated forms for the two lysine-less mutants Tax-1 K1-10R and Tax-2 K1-10iR. Similar patterns of sumoylated forms for wild type Tax-1 and Tax-2B were also observed (Figure 2B). Interestingly, although mutations of lysine K7 and K8 completely abolished sumoylation of mutant Tax-1 K7-8R, as previously reported [22], the equivalent mutant Tax-2 K7-8R still maintained 81% of sumoylation as compared to wild type Tax-2B. Moreover, Tax-2 K3ii-8R, with all 6 central lysine residues mutated to arginines, still had a detectable level of sumoylation (7%). As expected, when mutants Tax-1 K1-10R and Tax-2 K1-10iR with substitutions of all lysine residues were analyzed, no sumoylated forms were detected. The presence of comparable levels of wild type and mutant Tax proteins was ascertained by Western blot analysis using an anti-6His antibody (Figure 2C). These results indicate that Tax-2B central lysine residues K3ii to K8 are the major targets for ubiquitination, as observed for the equivalent K4 to K8 lysines of Tax-1. Interestingly, the sumoylation status of Tax-2B mutants was different from those of corresponding Tax-1 mutants. The fact that Tax-2 K7-8R sumoylation was reduced to 81% as compared to wild type Tax-2B indicates that lysines K7 and K8 are at least partly involved in Tax-2B sumoylation. A further reduction to 7% of wild type Tax-2B sumoylation is observed for mutant K3ii-8R indicating that the 4 lysines K3ii to K6 can also be sumoylated. Thus, contrary to Tax-1, lysine residues other that K7 and K8 can be sumoylated in Tax-2B.

### Ubiquitination and sumoylation deficiency alters the intracellular localization of both Tax-1 and Tax-2B

**Figure 3:**
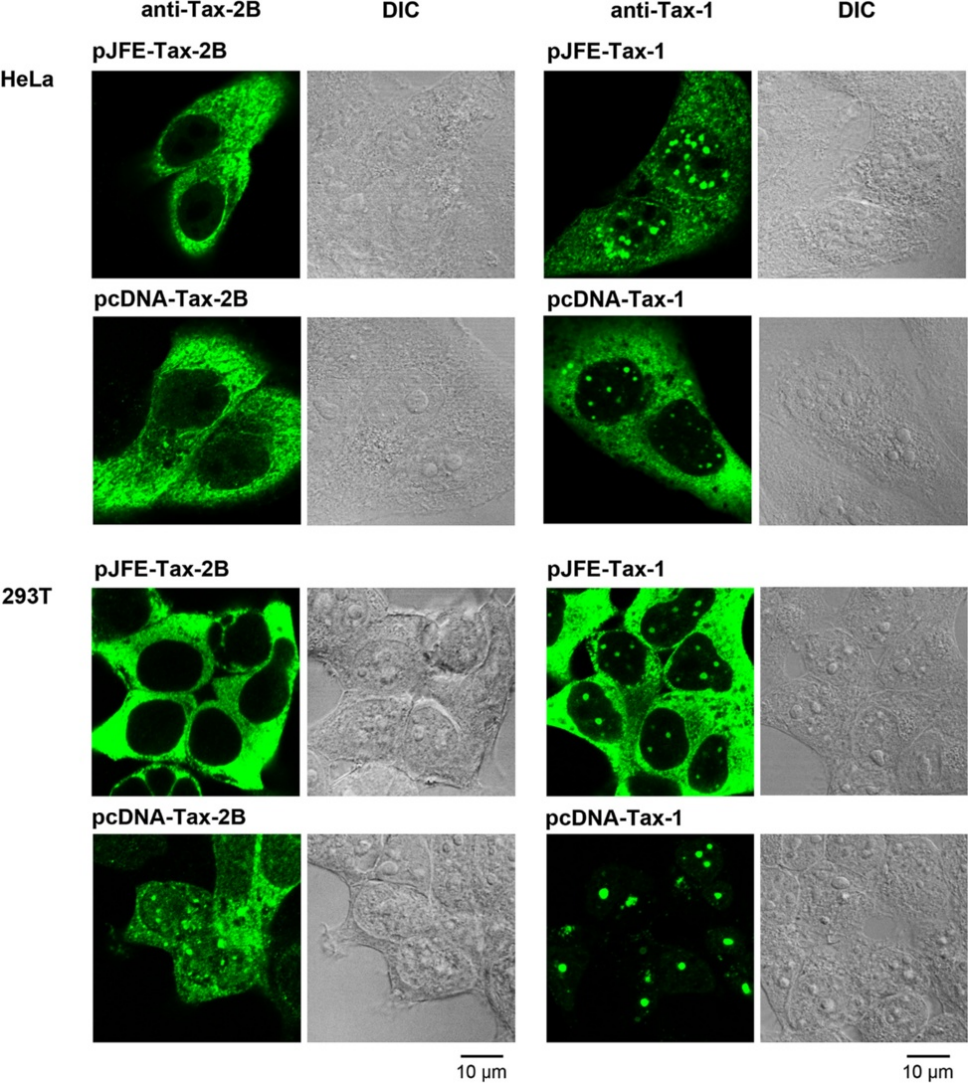
**The intracellular distribution of Tax**-**2B depends on the level and cellular context for expression. **HeLa or 293T cells were transfected with high level (pJFE) or low level (pcDNA) expression vectors for either Tax-1 or Tax-2B and analyzed by immunofluorescence staining and confocal microscopy with anti-Tax-1 IgG2a monoclonal antibody or anti-Tax-2B rabbit polyclonal antibody followed by anti-rabbit or anti-mouse IgG antibodies conjugated to Dylight 488. DIC, differential inference contrast.

The following series of experiments were designed to shed light on the controversy regarding the specifics of the intracellular distribution for Tax-1 and Tax-2B. First, we developed an anti-Tax-2B rabbit polyclonal antibody (cf Methods, Additional file 1). Second, the Tax-2B and Tax-1 genes were cloned in both pcDNA6.2 and pJFE14 expression vectors. We determined that cells transfected with pJFE-Tax vectors express 20 times more Tax proteins than cells transfected with the pcDNA-Tax vectors (data not shown). Third, we analyzed the intracellular distribution of Tax-2B with respect to Tax-1 by immunofluorescence staining using the newly developed anti-Tax-2B antibody in HeLa or 293T cells transfected with either the high level (pJFE) or the low level (pcDNA) expression vectors (Figure 3).

The results indicated that Tax-2B had a punctate nuclear distribution reminiscent of the Tax-1 nuclear bodies only in 293T cells transfected with pcDNA-Tax-2B vector. Either high and low level expression of Tax-2B in HeLa cells or high level expression in 293T cells strongly limited detection of Tax-2B in the nucleus and resulted in its accumulation in the cytoplasm. In contrast, Tax-1 NBs were observed in all tested conditions. Note that expression of Tax-1 using the low level expression vector in 293T cells resulted in undetectable Tax-1 in the cytoplasm and its concentration in Tax NBs in the majority of the cells. These results suggest that the balance between the cytoplasmic and nuclear distribution is more dependent upon the level and cellular background of expression for Tax-2B than for Tax-1.

**Figure 4:**
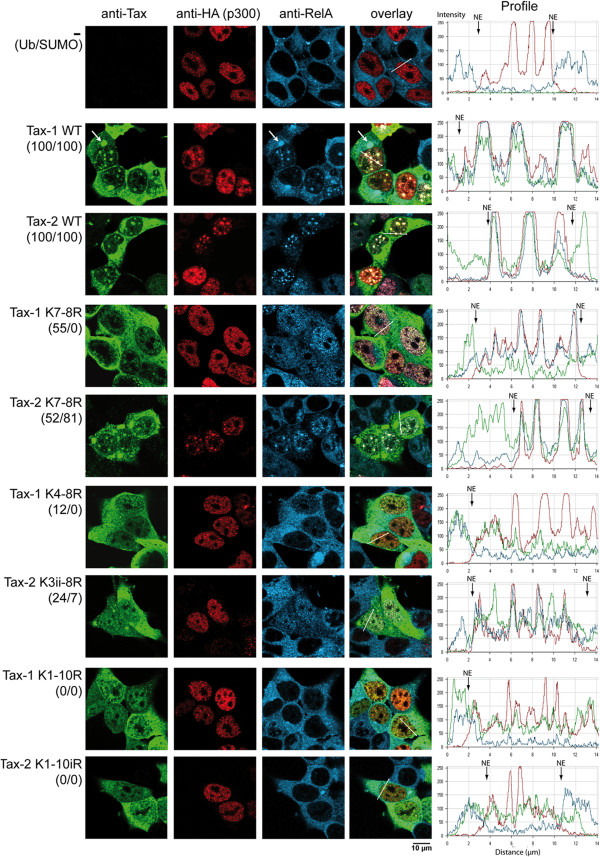
**Ubiquitination and sumoylation deficiency alters the intracellular localization of both Tax-1 and Tax-2B. **293T cells were cotransfected with vectors expressing wild type or mutant Tax-1 or Tax-2B and the transcriptional coactivator p300-HA. The cells were fixed, stained by triple immunofluorescence staining and analyzed by laser scanning confocal microscopy. Staining of Tax-1 expressing cells was with anti-Tax-1 IgG2a monoclonal antibody, anti-HA rabbit polyclonal antibody for the detection of p300-HA and anti-RelA IgG1 monoclonal antibody. For cells expressing Tax-2B, anti-Tax-2B rabbit polyclonal antibody and anti-HA monoclonal IgG2a antibody for the detection of p300-HA and the anti-RelA IgG1 monoclonal antibody were used. The secondary antibodies were goat anti-mouse IgG2a conjugated to Dylight 488, goat anti-rabbit IgG conjugated to Dylight 549 and goat anti-mouse IgG1 conjugated to Dylight 649. The profiles of the intensity of the fluorescence staining along lines crossing the nuclei are shown in the right panels (NE: nuclear envelope). The percentages of ubiquitination and sumoylation obtained in Figure 2 are indicated for each mutant (Ub/SUMO). White arrows point to cytoplasmic structures that contain Tax-1 and RelA at the boundary of the nucleus.

Because of the differences described in the preceding paragraph showing parameters that impact on Tax-2B distribution in the nucleus, we tested the intracellular localization of lysine to arginine substitution Tax-2B mutants in 293T cells transfected with pcDNA vectors expressing wild type or mutant Tax-2B proteins. Colocalization of Tax-1 with the RelA subunit of NF-κB and the transcriptional coactivator p300 in NBs is critical for Tax-1-mediated activation of the NF-κB pathway [27]. Therefore, we compared the intracellular localization of Tax proteins, as well as RelA and p300, by triple immunofluorescence staining in 293T cells cotransfected with vectors expressing wild type or mutant Tax proteins and p300-HA (Figure 4).

In cells that did not express Tax proteins, overexpressed p300-HA was detected only in the nucleus and displayed a speckled distribution, whereas endogenous RelA was only detected in the cytoplasm, as previously reported [27]. As indicated by the intensity of the fluorescence staining along a line drawn across the nucleus, wild type Tax-1 or Tax-2B concentrated within punctate nuclear structures in which both p300 and RelA were recruited. The punctate nuclear structures containing Tax-1, RelA and p300 were previously identified as Tax NBs by electron microscopy analysis [26]. It is interesting to note that, although both wild type Tax proteins were also detected in the cytoplasm, Tax-1, but much less frequently Tax-2B, concentrated with RelA in cytoplasmic structures at the boundary of the nucleus (highlighted by arrows in Figure 4). This observation will be further discussed below.

Remarkably, the Tax-2 K7-8R mutant, which maintained 52% of ubiquitination and 81% of sumoylation with respect to wild type Tax-2B, formed nuclear punctate structures in which p300 and RelA colocalized. This is in marked contrast with the equivalent ubiquitinated (55%), but non-sumoylated Tax-1 K7-8R mutant, which was deficient for formation of nuclear bodies but was able to induce the migration of RelA to the nucleus in a majority of the cells, as previously demonstrated [22]. Furthemore, Tax-2 K3ii-8R was still partly sumoylated and both RelA and p300 concentrated in nuclear punctate structures in a significant fraction of the cells, contrary to what was observed for the equivalent ubiquitination and sumoylation deficient Tax-1 K4-8R mutant.

Finally, the ubiquitination and sumoylation deficient Tax-1 K1-10R and Tax-2 K1-10iR mutants had diffuse distributions in the cytoplasm and the nucleus and did not induce the translocation of RelA to the nucleus. We also observed that endogenous p300 colocalized with Tax-2B in punctate nuclear structures, as demonstrated above for overexpressed p300 (Additional file 2). These results established a direct correlation between ubiquitination and translocation of RelA to the nucleus as well as between sumoylation and formation of punctate nuclear structures in which RelA and p300 were recruited for both Tax-1 and Tax-2B.

### Activation of gene expression via the NF-κB pathway correlates with the sumoylation and ubiquitination status of both Tax-1 and Tax-2B

**Figure 5:**
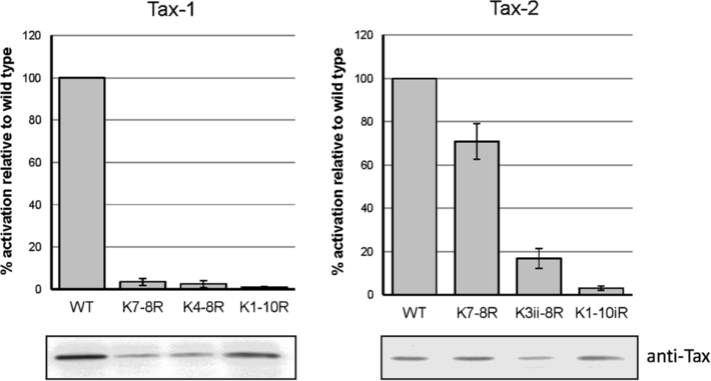
**Activation of gene expression via the NF-κB pathway correlates with Tax-1 and Tax-2B ubiquitination and sumoylation status. **293T cells were cotransfected with 50 ng of vectors expressing wild type or mutant Tax-1 or Tax-2B and 250 ng of NF-κB-Luc reporter plasmid. Fifty ng of the phRG-TK Renilla Luciferase vector were added to the reaction to normalize for transfection efficiency. Cells were lysed and the luciferase activity was measured. Mutant Tax-1 and Tax-2B activities were expressed as percentages of WT Tax activities. The values reported are the averages of three independent experiments. Comparable levels of wild type and mutant Tax proteins in the lysates were ascertained by Western blot analysis using Tax-1 and Tax-2B antibodies.

We then investigated the relevance of modification by ubiquitination and sumoylation in Tax-2B-mediated activation of the NF-κB pathway. We analyzed the capacities of wild type and mutant Tax-2B to activate gene expression from a construct expressing the luciferase reporter gene under the control of a promoter containing κB binding sites (NF-κB-luc reporter construct). The results were compared to those obtained for the equivalent wild type and mutant Tax-1 proteins (Figure 5). Notable differences between Tax-1 and Tax-2B emerged. Tax-1 K7-8R and K4-8R mutants were defective for NF-κB activation, thus confirming our previous findings [22], whereas the homologous Tax-2 K7-8R and K3ii-8R mutants maintained 70 and 18% of NF-κB activation, respectively. Both Tax-1 and Tax-2B mutants with all lysine residues substituted by arginines were defective for activation of gene expression via the NF-κB pathway. The expression level of each construct was controlled by detection with anti-Tax-1 and anti-Tax-2B antibodies. The fact that both Tax-2 K7-8R and K3ii-8R mutants were at least partly sumoylated and concentrated with RelA and p300 in nuclear punctate structures contrary to the equivalent Tax-1 mutants, clearly establishes a correlation between the sumoylation status of Tax-1 and Tax-2B mutants, their nuclear punctate distribution and their capacities to activate the NF-κB pathway.

### Fusion of ubiquitin or SUMO-1 to the C-terminus of wild type or K1-10iR mutant Tax-2B modifies their intracellular localization

**Figure 6:**
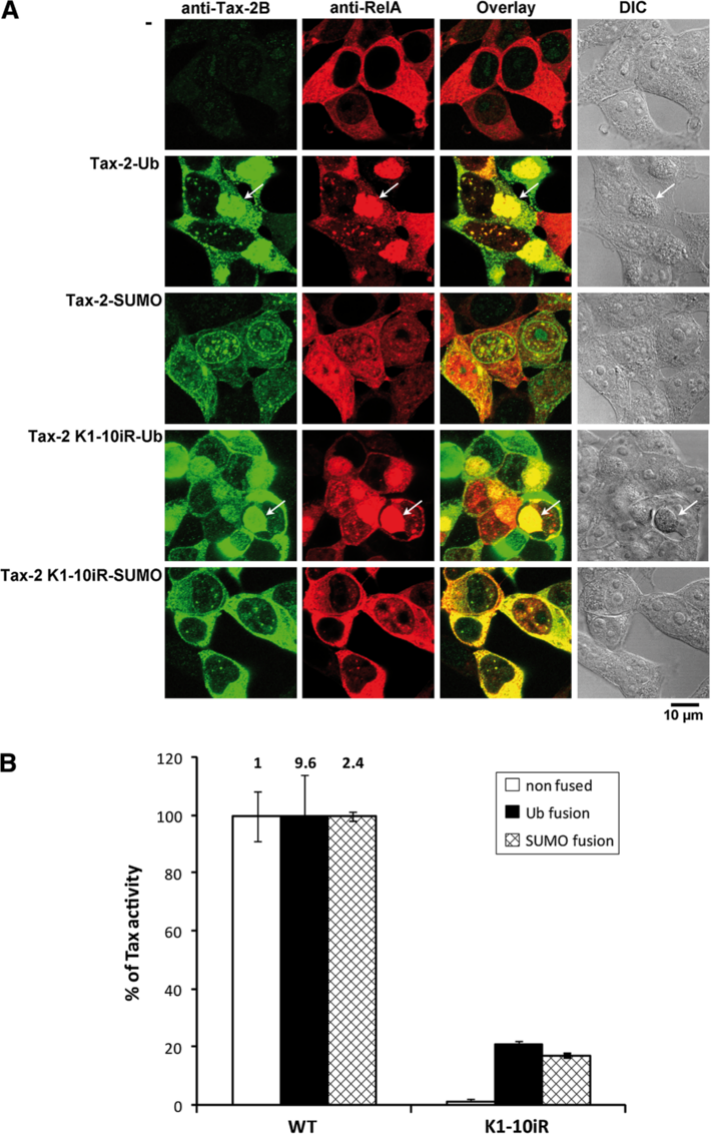
**Fusion of ubiquitin or SUMO**-**1 to the C**-**terminus of wild type or K1**-**10iR mutant modifies their intracellular localization and increases NF-κB activity. **(**A**) 293T cells were cotransfected with vectors expressing wild type or K1-10iR Tax-2B mutant, fused to ubiquitin (Tax-2-Ub and Tax-2 K1-10iR-Ub) or SUMO-1 (Tax-2-SUMO and Tax-2 K1-10iR-SUMO). The cells were fixed and stained by dual immunofluorescence staining with anti-Tax-2B rabbit polyclonal antibody and anti-RelA IgG1 monoclonal antibody. The secondary antibodies were goat anti-rabbit IgG conjugated to Dylight 549 and goat anti-mouse IgG1 conjugated to Dylight 649. The images were collected using a laser scanning confocal microscope. White arrows point to cytoplasmic structures at the boundary of the nucleus in which Tax-2-Ub and Tax-2 K1-10iR-Ub fusions colocalize with RelA. DIC, differential inference contrast. (**B**) 293T cells were cotransfected with 50 ng of vectors expressing wild type or K1-10iR mutant Tax-2B fused or not to ubiquitin or SUMO-1 and 250 ng of the NF-κB-Luc reporter construct. 50 ng of the phRG-TK Renilla Luciferase vector was added to normalize for transfection efficiency. Cells were lysed and the luciferase activity was measured in each extracts. The activity of each mutant fusion is expressed as percentages relatively to the equivalent wild type Tax-2-Ub or Tax-2-SUMO fusions. The values were normalized for equal quantity of Tax proteins in each extract. The values reported are the averages of three independent experiments.

To determine whether ubiquitination and sumoylation had direct consequences on Tax-2B intracellular localization and transcriptional activity, we constructed fusions of ubiquitin or SUMO-1 to the C-terminus of the lysine-less Tax-2 K1-10iR mutant (Tax-2 K1-10iR-Ub and Tax-2 K1-10iR-SUMO). The equivalent fusions of wild type Tax-2B (Tax-2-Ub and Tax-2-SUMO) were constructed as controls. 293T cells transfected with the various fusions were analyzed by dual immunofluorescence staining and confocal microscopy using anti-Tax-2B and anti-RelA antibodies (Figure 6A).

Comparison of the results in Figure 6A with those obtained in Figure 4 indicates that both wild type and K1-10iR mutant Tax-2-Ub fusions colocalized in the majority of the cells with RelA in prominent cytoplasmic structures localized at the boundary of the nucleus (indicated by arrows in Figure 6A), contrary to their unfused versions (see Figure 4). Immunostaining of GM130, a Golgi matrix protein, and IKKγ, the regulatory subunit of the IKK complexes, indicated that the cytoplasmic structures containing the wild type Tax-2-Ub fusion were closely associated with the Golgi apparatus and concentrated IKKγ, in addition to RelA (Additional file 3). These structures are reminiscent of the Tax-1/RelA/IKKγ-containing cytoplasmic “hot spots” previously identified in Tax-1 and Tax-1-Ub expressing cells (see Figure 4 above and [22,23]). Interestingly, the Tax-2 K1-10iR-Ub fusion was able to induce the migration of RelA to the nucleus, contrary to the unfused Tax-2 K1-10iR mutant.

The wild type Tax-2-SUMO fusion was predominantly distributed in prominent nuclear foci that included RelA, and this fusion was less detected in the cytoplasm and did not concentrate in cytoplasmic “hot spots”, as compared to the Tax-2-Ub fusion. Furthermore, the Tax-2 K1-10iR-SUMO fusion was distributed in nuclear foci, contrary to the unfused K1-10iR mutant. This fusion was able to induce the migration of RelA to the nucleus, suggesting that fusion of SUMO-1 to the modification deficient K1-10iR mutant can compensate the lack of ubiquitination for activation of the IKK complexes in the cytoplasm. Similar compensation of ubiquitination by SUMO-1 fusion for induction of RelA migration to the nucleus was previously observed for Tax-1 K4-8R-SUMO fusion [22]. Thus, fusion of ubiquitin to the C-terminus of the Tax-2 K1-10iR mutant determined the targeting of the fusion to Golgi-associated structures containing RelA and IKKγ, whereas fusion of SUMO-1 favored the formation of nuclear foci containing RelA. In addition, both fusions were able to induce the translocation of RelA to the nucleus.

### Fusion of ubiquitin or SUMO-1 to the C-terminus of the K1-10iR Tax-2B mutant restores NF-κB activity

Finally, we tested whether fusion of ubiquitin or SUMO-1 to wild type or K1-10iR Tax-2B resulted in altered capacities to activate the NF-κB pathway. Figure 6B indicates that the wild type Tax-2-Ub and Tax-2-SUMO fusions had a markedly increased ability to activate the NF-κB pathway as compared to the unfused wild type Tax-2B control (9.6 and 2.4 fold increase, respectively). In addition, although the unfused mutant K1-10iR was deficient for activation of the NF-κB pathway (1% of wild type unfused Tax-2B), ubiquitin or SUMO-1 fusion to this mutant increased its transcriptional activity by 21 and 17 fold as compared to their wild type Tax-2-Ub and Tax-2-SUMO controls, respectively. These results strongly suggest that both ubiquitination and sumoylation are directly involved in Tax-2B-mediated activation of the NF-κB pathway.

## Discussion

We have compared the differences between the Tax proteins of HTLV-1 and HTLV-2 that are involved in their capacity to activate the NF-κB pathway. Tax-1-mediated activation of the NF-κB pathway is regulated by ubiquitination and sumoylation of lysine residues in its central domain and these modifications control Tax-1 intracellular localization. We thus compared the modification patterns of the two proteins by using a panel of lysine to arginine substitution mutants. First, mutations of the lysine residues in the central domain of the two proteins (K4 to K8 for Tax-1 and K3ii to K8 for Tax-2B) resulted in a significant decrease of the ubiquitination level (12 and 24%, respectively), indicating that these central lysine residues are major targets for ubiquitination. Second, sumoylation, which is restricted to lysines K7 and K8 for Tax-1, is targeted to all lysines of the central domains in Tax-2B since only mutation of lysines K3ii to K8 led to a marked reduction of Tax-2 sumoylation level (7%). As expected, substitution of all lysine residues in the two proteins led to ubiquitination and sumoylation deficiency.

Thus, the lysine usage for sumoylation differs between Tax-1 and Tax-2B, which might indicate that the two proteins have different tridimensional folding of their central domain leading to differential interaction with the sumoylation machinery. The differential lysine usage for sumoylation has clear consequences on the intracellular localization and transcriptional activity as indicated by the observations that the sumoylation deficient Tax-1 K7-8R mutant does not assemble nuclear bodies and has no transcriptional activity, contrary to the equivalent Tax-2 K7-8R mutant.

Our results shed light on the differences reported in the literature about Tax-2B distribution in the nucleus. The comparative intracellular localization of Tax-1 and Tax-2B has been thoroughly investigated [29-31], but with controversial results. These studies support the idea that Tax-2B is more predominant in the cytoplasm than Tax-1 and that Tax-1 displays a striking punctate distribution in the nucleus. By using various experimental settings, we find out that overexpression of Tax-2B prevents its detection in the nucleus and leads to its accumulation in the cytoplasm. The level of expression enabling efficient transport to the nucleus appears to depend on the cellular background used for expression.

The comparative analysis of the ubiquitination pattern of Tax-1 and Tax-2B points to a reduced representation of the Tax-2B mono-ubiquitinated forms relatively to Tax-1 (Figure 2), which might explain the differential distribution of Tax-2B and Tax-1 in the cytoplasm and the reduced transport of Tax-2B to the nucleus. This suggestion is supported by the observation that fusion of ubiquitin, which mimics mono-ubiquitination, favors the concentration of Tax-2B (and Tax-1) in Golgi-associated structures at the boundary of the nucleus. The contribution of these structures in Tax-1-mediated activation of IKK complexes has been established [23,24]. However, our previous observations suggest that these cytoplasmic structures might also be involved in the translocation of both Tax-1 and RelA to the nucleus. Whether limited mono-ubiquitination controls the balance between nuclear and cytoplasmic Tax-2B, displacing the equilibrium toward the cytoplasm, and whether the cellular background and the level of expression affect mono-ubiquitination of Tax-2B will require further investigations. In addition, more work is required to determine whether specific differences in the modification patterns of Tax-1 and Tax-2B, including lysine usage for sumoylation and level of mono-ubiquitination, might impact their oncogenic potential.

Besides these differences, our work demonstrates that both ubiquitination and sumoylation control Tax-2B intracellular localization and ability to activate the NF-κB pathway. Polyubiquitination, and specifically K63-branched polyubiquitination, is a critical modifier of several effectors of the NF-κB phosphorylation cascade [32] as well as of Tax-1. Polyubiquitination of Tax-1 was demonstrated to determine the targeting of IKKγ to Golgi-associated structures involved in the activation of the IKK complexes [22-25,33]. Similarly, Tax-2B polyubiquitination could play a role in the release of RelA sequestration by NF-κB inhibitors in the cytoplasm and its subsequent migration to the nucleus. This conclusion is supported by the correlation between the loss of ubiquitination of the lysine-less Tax-2 K1-10iR mutant and its inability to induce the migration of RelA to the nucleus. Furthermore, the striking increase in transcriptional activity resulting from fusion of ubiquitin to the C-terminus of both wild type and the lysine-less Tax-2B mutant correlated with formation of cytoplasmic structures associated with the Golgi apparatus and the recruitment of RelA and IKKγ to these structures.

Polysumoylation is involved in formation of nuclear bodies by many cellular and viral proteins, including Tax-1 [22,25,34]. The direct correlation between the level of sumoylation of Tax-2B lysine mutants and SUMO-1 fusion to the lysine-less mutant, their ability to form punctate nuclear structures that included RelA and p300 and to activate gene expression via the NF-κB pathway support the idea that sumoylation and targeting to punctate nuclear structures play critical roles in Tax-2B transcriptional activity.

The concept that SUMO modification of Tax proteins plays an active role in activation of the NF-κB pathway [22,25,28-30] reviewed in [20,21] is challenged by a new study by Bonnet *et al*. [35]. The study shows that a Tax-1 mutant (P79AQ81A) with sumoylation that is only 23% of wild type Tax-1 and reduced ability to form detectable NBs is still transcriptionally active. Such results might be reconciled with our observations if the Tax-1 mutant P79AQ81A maintains a level of sumoylation sufficient for threshold activity. A threshold governing the relationship between sumoylation and transcriptional activity is in line with the well known observation that the biological consequences of SUMO conjugation is not proportional to the small fraction of substrate that is modified [36]. In addition, detection of NBs at the resolution level of light microscopy might require a sumoylation threshold higher than that required for transcriptional activity. If we assume that Tax NBs are sites of transcription, it is interesting to note that the estimated size of transcription factories is 70-80 nm [37], which is considerably under the resolution of immunofluorescence staining and confocal microscopy. Thus, detection of Tax NBs might depend on overexpression of Tax or Tax-SUMO fusion, but might be limited in cells expressing Tax-1 mutant P79AQ81A or in HTLV-1 infected T-lymphocytes. These suggestions are supported by the observation that Tax-1 mutant K7R, which has a reduced sumoylation status (30% of wild type Tax-1), gives both NB formation and transcriptional activity similar to the phenotype of mutant P79AQ81A [35]. In a recent paper, Xiao [38] concludes that the studies of Bonnet *et al*. [35] cannot rule out the possibility that Tax sumoylation and/or nuclear bodies may be involved in the transcriptional regulation of some specific NF-κB target genes.

This study supports the idea that both Tax-1 and Tax-2B need to be targeted to specific subcellular domains to achieve NF-κB activation and broadens our model depicting the cytoplasmic and nuclear steps involved in Tax-1-mediated activation of the NF-κB pathway [22]. In addition, Tax-1 and Tax-2B have common partners both in the cytoplasm and nucleus. The results of this study, showing that the punctate nuclear structures assembled by Tax-2B include the RelA subunit of NF-κB and the p300 transcriptional coactivator, indicate a common property with the previously identified Tax-1 NBs. This work is also consistent with our previous studies showing that Tax-2B and Tax-1 colocalize in the cytoplasm with cellular effectors of the NF-κB pathway activation pathway RelA, IKKγ and TAB2 [30].

## Conclusions

A huge body of evidence indicates that the transforming activity of Tax-1 depends on its capacity to activate the NF-κB pathway. However, this property is most likely necessary but not sufficient for cellular transformation. Since Tax-2B activates the NF-κB pathway to similar level and via similar mechanisms, the lack of malignancy of HTLV-2 might result from other functional properties that differ between Tax-1 and Tax-2B such as activation of the non-canonical NF-κB pathway, interaction with PDZ-domain proteins and/or other properties yet to be revealed.

## Methods

### Plasmid constructs

The alignment and nomenclature of the lysine residues in Tax-1 and Tax-2B are shown in Figure 1. The plasmid constructs expressing the wild type or lysine to arginine substitution mutants K7-8R, K4-8R and K1-10R of Tax-1 fused to a C-terminal 6-histidine tag were as previously described [22]. The pcDNA-Tax-1 vector obtained by replacing the sequence coding for Tax-1F in pcDNA6.2 Tax-1F expression vector [29] by the sequence coding for Tax-1. The Tax-2B cDNA was cloned into both the pJFE14 vector [39] and the pcDNA6.2/N expression vector (Invitrogen Corporation; Carlsbad, Ca, USA) using *Eco*RI restriction sites. A similar construct generated pcDNA6.2-Tax-2-6His. This construct was submitted to site-directed mutagenesis to generate the plasmids expressing the Tax-2-6His K7-8R, K3ii-8R and K1-10iR mutants using the Stratagene mutagenesis kit. The vectors for the expression of wild type or K1-10iR mutant Tax-2B fusions to ubiquitin or SUMO-1 were obtained by PCR amplification of the ubiquitin or SUMO-1 coding sequence followed by a 6 histidine tag and the cloning of the resulting fragments at the 3’ end of the Tax coding sequence in vectors pcDNA6.2-Tax-2 and pcDNA6.2-Tax-2 K1-10iR. The di-glycine motif involved in the conjugation reaction at the end of the ubiquitin and SUMO-1 sequences were replaced by Gly-Ala to prevent conjugation of the fusions to other substrates. Each mutant or fusion was submitted to nucleotide sequence analysis to verify the correct nucleotide sequence and mutations. The vectors expressing ubiquitin or SUMO-1 tagged with the hemagglutinin epitope of the Influenza virus (HA) at their N-terminus have been previously described [40,41]. The vector for expression of p300-HA has been previously described [42]. The reporter plasmid NF-κB LTR-luc was purchased from Stratagene.

### Cell culture and transfection

293T and HeLa cells were maintained in Dulbecco’s modified Eagle’s medium (DMEM) supplemented with 2 mM L-Glutamine, 10% foetal calf serum (FCS), 1% penicillin-streptomycin and 1 mM sodium pyruvate (Gibco) and transfected using the Transit LT1 reagent (Mirus Bio LLC) according to the manufacturer’s instructions.

### Antibodies

The Tax-2B polyclonal antibody was obtained by immunizing rabbits with a peptide sequence representing the 20 C-terminal amino acids of Tax-2B (AAQGESSTQKVRPSHTNNPK). The antibody was further purified by affinity chromatography against the immobilized peptide. Its binding specificity was determined by Western Blot and immunoprecipitation analyses (Additional file 1). The anti-Tax-1 monoclonal antibody from hybridoma 168-A51 was obtained from the AIDS research and Reagent Program, National Institutes of Health. The anti-RelA IgG1 mouse monoclonal antibody (F-6), the anti-HA IgG2a monoclonal antibody (F-7), the anti-HA rabbit polyclonal antibody (Y-11) and the anti-IKKγ IgG1 monoclonal antibody (B-3) were purchased from Santa Cruz Biotechnology. The anti-p300 IgG1 mouse monoclonal antibody (RW128) was from Upstate. The anti-pentaHis mouse antibody was from Qiagen. The anti-GM130 was from BD Biosciences. The secondary antibodies used for immunofluorescence staining were Dylight 488-conjugated goat anti-mouse IgG2a, Dylight 549- or 488-conjugated goat anti-rabbit IgG and Dylight 649-conjugated goat anti-mouse IgG1 from Jackson ImmunoResearch.

### Luciferase assays

Tax-1- and Tax-2B-mediated activation of gene expression via the NF-κB pathway was assayed by dual luciferase assays using the NF-κB-luc reporter construct. 293T cells (1.25 x 10^5^) were transfected into 12 wells with 250 ng of NF-κB-luc reporter plasmids, 50 ng of each of the different Tax-1 or Tax-2B constructs, and 50 ng of phRG-TK Renilla Luciferase vector (Promega) used to monitor transfection efficiency. Cells were lysed and tested for luciferase activity with a luminometer TD-20/20 (Turner Designs) using the Dual-luciferase reporter assay system (Promega).

### Ni-NTA pulldown and western blot analysis

293T cells were lysed 24 h after transfection and Ni-NTA pulldown was done as described previously [22]. The purified proteins were analyzed by electrophoresis on 4-12% Bis-Tris NuPAGE gel (Invitrogen), transferred to Hybond ECL Nitrocellulose membrane (Amersham Pharmacia Biotech), and immunoblotted with primary and corresponding secondary antibodies. Detection was by Lumi-Light Western Blotting Substrate (Roche) or Chemi-luminescent Peroxidase Substrate (Sigma Aldrich). Detection and quantitation of chemiluminescent signals were performed with the Chemi-Smart 5000 and Bio-1D software (Vilber Lourmat) or Autochemi and Gel Pro Analyzer (UVP BioImaging Systems).

### Immunofluorescence staining

Cells cultured on glass coverlips were transfected with expression plasmids for 24 h, fixed in Immunohistofix (Gentaur) at room temperature for 10 minutes followed by incubation in methanol for 6 minutes at -20°C and incubated with the primary antibodies overnight at 4°C. After washing, the specimens were incubated for 2 h at room temperature with the secondary antibodies. Analysis was with a laser scanning confocal microscope (LSM 510, Zeiss) equipped with a 63x objective and light source wavelengths of 488, 543 and 633 nm.

## Competing interests

The authors declare that they have no competing interests.

## Authors’ contributions

MT, JL, GDG, ASR, OM, ED and CS performed experiments. FB, UB and MGR conceived the study, analyzed the data and wrote the paper. All authors read and approved the final manuscript.

## Supplementary Material

Additional file 1 **Specificity of the purified anti-Tax-2B rabbit polyclonal antibody. **Total cell lysates (30 μg) from 293T cells expressing either Tax-2B or Tax-1 were analyzed (A) by Western blotting with the purified rabbit polyclonal anti-Tax-2B antibody developed in this work (1:2000 dilution). (B) These lysates were also immunoprecipitated with the same anti-Tax-2B antibody. The input represents 1/10 of the immunoprecipitated lysate. Click here for file

Additional file 2 **Tax-1 and Tax-2B colocalize with endogenous p300 in nuclear punctate structures. **293T cells were either transfected or not with the vectors expressing Tax-1 or Tax-2B. The cells were fixed and analyzed by dual immunofluorescence staining with the anti-Tax-1 IgG2a monoclonal antibody or the anti-Tax-2B rabbit polyclonal antibody and an anti-p300 IgG1 monoclonal antibody. The secondary antibodies were goat anti-mouse IgG2a conjugated to Dylight 488, goat anti-mouse IgG1 antibody conjugated to Dylight 649 and goat anti-rabbit IgG antibody conjugated to Dylight 549. The images were collected using a laser scanning confocal microscope. DIC, differential inference contrast. Click here for file

Additional file 3 **The wild type Tax-2B fusion to ubiquitin colocalizes with IKKγ in prominent cytoplasmic structures closely associated with the Golgi apparatus. **293T cells transfected or not with the vector expressing the Tax-2-Ub fusion were fixed and analyzed by dual immunofluorescence staining with anti-Tax-2B rabbit polyclonal antibody and (A) anti-GM130 IgG1 mouse monoclonal antibody or (B) anti-IKKγ IgG1 mouse monoclonal antibody. The secondary antibodies were goat anti-mouse IgG1 antibody conjugated to Dylight 649 and goat anti-rabbit IgG antibody conjugated to Dylight 549. The images were collected using a laser scanning confocal microscopy. DIC, differential inference contrast. Click here for file

## References

[B1] Matsuoka M Jeang KT Human T-cell leukaemia virus type 1 (HTLV-1) infectivity and cellular transformation Nat Rev Cancer 2007 7 270 280 10.1038/nrc2111 17384582

[B2] Araujo A Hall WW Human T-lymphotropic virus type II and neurological disease Ann Neurol 2004 56 10 19 10.1002/ana.20126 15236397

[B3] Lewis MJ Sheehy N Salemi M Vandamme AM Hall WW Comparison of CREB- and NF-kappaB-mediated transactivation by human T lymphotropic virus type II (HTLV-II) and type I (HTLV-I) tax proteins Virology 2002 295 182 189 10.1006/viro.2002.1357 12033776

[B4] Grassmann R Aboud M Jeang KT Molecular mechanisms of cellular transformation by HTLV-1 Tax Oncogene 2005 24 5976 5985 10.1038/sj.onc.1208978 16155604

[B5] Marriott SJ Semmes OJ Impact of HTLV-I Tax on cell cycle progression and the cellular DNA damage repair response Oncogene 2005 24 5986 5995 10.1038/sj.onc.1208976 16155605

[B6] Giam CZ Jeang KT HTLV-1 Tax and adult T-cell leukemia Front Biosci 2007 12 1496 1507 10.2741/2163 17127397

[B7] Boxus M Willems L Mechanisms of HTLV-1 persistence and transformation Br J Cancer 2009 101 1497 1501 10.1038/sj.bjc.6605345 19861996 PMC2778510

[B8] Boxus M Willems L How the DNA damage response determines the fate of HTLV-1 Tax-expressing cells Retrovirology 2012 9 2 10.1186/1742-4690-9-2 22221708 PMC3283471

[B9] Zane L Sibon D Jeannin L Zandecki M Delfau-Larue MH Gessain A Tax gene expression and cell cycling but not cell death are selected during HTLV-1 infection in vivo Retrovirology 2010 7 17 10.1186/1742-4690-7-17 20222966 PMC2846874

[B10] Mahieux R Pise-Masison CA Nicot C Green P Hall WW Brady JN Inactivation of p53 by HTLV type 1 and HTLV type 2 Tax trans-activators AIDS Res Hum Retroviruses 2000 16 1677 1681 10.1089/08892220050193137 11080809

[B11] Peloponese JM Jr Kinjo T Jeang KT Human T-cell leukemia virus type 1 Tax and cellular transformation Int J Hematol 2007 86 101 106 10.1532/IJH97.07087 17875521

[B12] Bertazzoni U Turci M Avesani F di Gennaro G Bidoia C Romanelli MG Intracellular localization and cellular factors interaction of HTLV-1 and HTLV-2 Tax proteins: similarities and functional differences Viruses 2011 3 541 560 10.3390/v3050541 21994745 PMC3185761

[B13] Higuchi M Tsubata C Kondo R Yoshida S Takahashi M Oie M Cooperation of NF-kappaB2/p100 activation and the PDZ domain binding motif signal in human T-cell leukemia virus type 1 (HTLV-1) Tax1 but not HTLV-2 Tax2 is crucial for interleukin-2-independent growth transformation of a T-cell line J Virol 2007 81 11900 11907 10.1128/JVI.00532-07 17715223 PMC2168800

[B14] Shoji T Higuchi M Kondo R Takahashi M Oie M Tanaka Y Identification of a novel motif responsible for the distinctive transforming activity of human T-cell leukemia virus (HTLV) type 1 Tax1 protein from HTLV-2 Tax2 Retrovirology 2009 6 83 10.1186/1742-4690-6-83 19761585 PMC2754985

[B15] Semmes OJ Majone F Cantemir C Turchetto L Hjelle B Jeang KT HTLV-I and HTLV-II Tax: differences in induction of micronuclei in cells and transcriptional activation of viral LTRs Virology 1996 217 373 379 10.1006/viro.1996.0126 8599225

[B16] Hirata A Higuchi M Niinuma A Ohashi M Fukushi M Oie M PDZ domain-binding motif of human T-cell leukemia virus type 1 Tax oncoprotein augments the transforming activity in a rat fibroblast cell line Virology 2004 318 327 336 10.1016/j.virol.2003.10.006 14972558

[B17] Rousset R Fabre S Desbois C Bantignies F Jalinot P The C-terminus of the HTLV-1 Tax oncoprotein mediates interaction with the PDZ domain of cellular proteins Oncogene 1998 16 643 654 10.1038/sj.onc.1201567 9482110

[B18] Xie L Yamamoto B Haoudi A Semmes OJ Green PL PDZ binding motif of HTLV-1 Tax promotes virus-mediated T-cell proliferation in vitro and persistence in vivo Blood 2006 107 1980 1988 10.1182/blood-2005-03-1333 16263794 PMC1895710

[B19] Van PL Yim KW Jin DY Dapolito G Kurimasa A Jeang KT Genetic evidence of a role for ATM in functional interaction between human T-cell leukemia virus type 1 Tax and p53 J Virol 2001 75 396 407 10.1128/JVI.75.1.396-407.2001 11119608 PMC113932

[B20] Lodewick J Lamsoul I Bex F Move or die: the fate of the Tax oncoprotein of HTLV-1 Viruses 2011 3 829 857 10.3390/v3060829 21994756 PMC3185767

[B21] Kfoury Y Nasr R Journo C Mahieux R Pique C Bazarbachi A The multifaceted oncoprotein Tax: subcellular localization, posttranslational modifications, and NF-kappaB activation Adv Cancer Res 2012 113 85 120 22429853 10.1016/B978-0-12-394280-7.00003-8

[B22] Lamsoul I Lodewick J Lebrun S Brasseur R Burny A Gaynor RB Exclusive ubiquitination and sumoylation on overlapping lysine residues mediate NF-kappaB activation by the human T-cell leukemia virus tax oncoprotein Mol Cell Biol 2005 25 10391 10406 10.1128/MCB.25.23.10391-10406.2005 16287853 PMC1291224

[B23] Harhaj NS Sun SC Harhaj EW Activation of NF-kappa B by the human T cell leukemia virus type I Tax oncoprotein is associated with ubiquitin-dependent relocalization of I kappa B kinase J Biol Chem 2007 282 4185 4192 17145747 10.1074/jbc.M611031200

[B24] Shembade N Harhaj NS Yamamoto M Akira S Harhaj EW The human T-cell leukemia virus type 1 Tax oncoprotein requires the ubiquitin-conjugating enzyme Ubc13 for NF-kappaB activation J Virol 2007 81 13735 13742 10.1128/JVI.01790-07 17942533 PMC2168884

[B25] Nasr R Chiari E El Sabban M Mahieux R Kfoury Y Abdulhay M Tax ubiquitylation and sumoylation control critical cytoplasmic and nuclear steps of NF-kappa B activation Blood 2006 107 4021 4029 10.1182/blood-2005-09-3572 16424386

[B26] Bex F McDowall A Burny A Gaynor R The human T-cell leukemia virus type 1 transactivator protein Tax colocalizes in unique nuclear structures with NF-kappaB proteins J Virol 1997 71 3484 3497 9094620 10.1128/jvi.71.5.3484-3497.1997 PMC191495

[B27] Bex F Yin MJ Burny A Gaynor RB Differential transcriptional activation by human T-cell leukemia virus type 1 Tax mutants is mediated by distinct interactions with CREB binding protein and p300 Mol Cell Biol 1998 18 2392 2405 9528808 10.1128/mcb.18.4.2392 PMC121497

[B28] Kfoury Y Setterblad N El Sabban M Zamborlini A Dassouki Z El Hajj H Tax ubiquitylation and SUMOylation control the dynamic shuttling of Tax and NEMO between Ubc9 nuclear bodies and the centrosome Blood 2011 117 190 199 10.1182/blood-2010-05-285742 20959607

[B29] Turci M Lodewick J Righi P Polania A Romanelli MG Bex F HTLV-2B Tax oncoprotein is modified by ubiquitination and sumoylation and displays intracellular localization similar to its homologue HTLV-1 Tax Virology 2009 386 6 11 10.1016/j.virol.2009.01.003 19195675

[B30] Avesani F Romanelli MG Turci M di Gennaro G Sampaio C Bidoia C Association of HTLV Tax proteins with TAK1-binding protein 2 and RelA in calreticulin-containing cytoplasmic structures participates in Tax-mediated NF-kappaB activation Virology 2010 408 39 48 10.1016/j.virol.2010.08.023 20875659

[B31] Meertens L Chevalier S Weil R Gessain A Mahieux R A 10-amino acid domain within human T-cell leukemia virus type 1 and type 2 tax protein sequences is responsible for their divergent subcellular distribution J Biol Chem 2004 279 43307 43320 10.1074/jbc.M400497200 15269214

[B32] Wertz IE Dixit VM Signaling to NF-kappaB: regulation by ubiquitination Cold Spring Harb Perspect Biol 2010 2 a003350 10.1101/cshperspect.a003350 20300215 PMC2829959

[B33] Gohda J Irisawa M Tanaka Y Sato S Ohtani K Fujisawa J HTLV-1 Tax-induced NFkappaB activation is independent of Lys-63-linked-type polyubiquitination Biochem Biophys Res Commun 2007 357 225 230 10.1016/j.bbrc.2007.03.125 17418100

[B34] Van Damme E Laukens K Dang TH Van Ostade X A manually curated network of the PML nuclear body interactome reveals an important role for PML-NBs in SUMOylation dynamics Int J Biol Sci 2010 6 51 67 20087442 10.7150/ijbs.6.51 PMC2808052

[B35] Bonnet A Randrianarison-Huetz V Nzounza P Nedelec M Chazal M Waast L Low nuclear body formation and tax SUMOylation do not prevent NF-kappaB promoter activation Retrovirology 2012 9 77 10.1186/1742-4690-9-77 23009398 PMC3476979

[B36] Hay RT SUMO: a history of modification Mol Cell 2005 18 1 12 10.1016/j.molcel.2005.03.012 15808504

[B37] Iborra FJ Pombo A McManus J Jackson DA Cook PR The topology of transcription by immobilized polymerases Exp Cell Res 1996 229 167 173 10.1006/excr.1996.0355 8986593

[B38] Xiao G NF-kappaB activation: Tax sumoylation is out, but what about Tax ubiquitination? Retrovirology 2012 9 78 10.1186/1742-4690-9-78 23009565 PMC3470980

[B39] Takebe Y Seiki M Fujisawa J Hoy P Yokota K Arai K SR alpha promoter: an efficient and versatile mammalian cDNA expression system composed of the simian virus 40 early promoter and the R-U5 segment of human T-cell leukemia virus type 1 long terminal repeat Mol Cell Biol 1988 8 466 472 2827008 10.1128/mcb.8.1.466-472.1988 PMC363152

[B40] Chiari E Lamsoul I Lodewick J Chopin C Bex F Pique C Stable ubiquitination of human T-cell leukemia virus type 1 tax is required for proteasome binding J Virol 2004 78 11823 11832 10.1128/JVI.78.21.11823-11832.2004 15479824 PMC523289

[B41] Tatham MH Jaffray E Vaughan OA Desterro JM Botting CH Naismith JH Polymeric chains of SUMO-2 and SUMO-3 are conjugated to protein substrates by SAE1/SAE2 and Ubc9 J Biol Chem 2001 276 35368 35374 10.1074/jbc.M104214200 11451954

[B42] Hanstein B Eckner R DiRenzo J Halachmi S Liu H Searcy B p300 Is a component of an Estrogen receptor coactivator complex Proc Natl Acad Sci U S A 1996 93 11540 11545 10.1073/pnas.93.21.11540 8876171 PMC38093

