# Supplementary figures and images for: Ubiquitination and sumoylation of the HTLV-2 Tax-2B protein regulate its NF-κB activity: a comparative study with the HTLV-1 Tax-1 protein
Source: Retrovirology. 2012 Dec 7;9:102. doi: 10.1186/1742-4690-9-102 (PMC3543174; doi:10.1186/1742-4690-9-102)

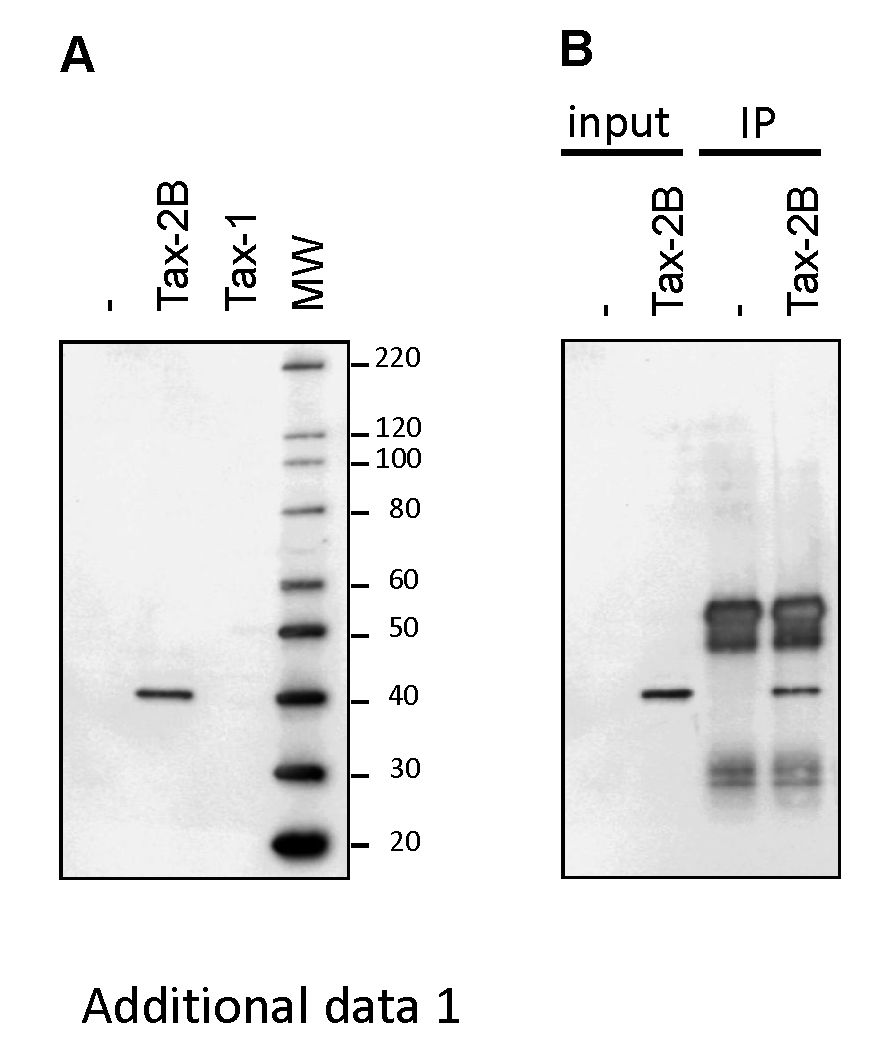

Supplement: Additional file 1 — Specificity of the purified anti-Tax-2B rabbit polyclonal antibody. Total cell lysates (30 μg) from 293T cells expressing either Tax-2B or Tax-1 were analyzed (A) by Western blotting with the purified rabbit polyclonal anti-Tax-2B antibody developed in this work (1:2000 dilution). (B) These lysates were also immunoprecipitated with the same anti-Tax-2B antibody. The input represents 1/10 of the immunoprecipitated lysate. [file 1742-4690-9-102-S1.tiff]

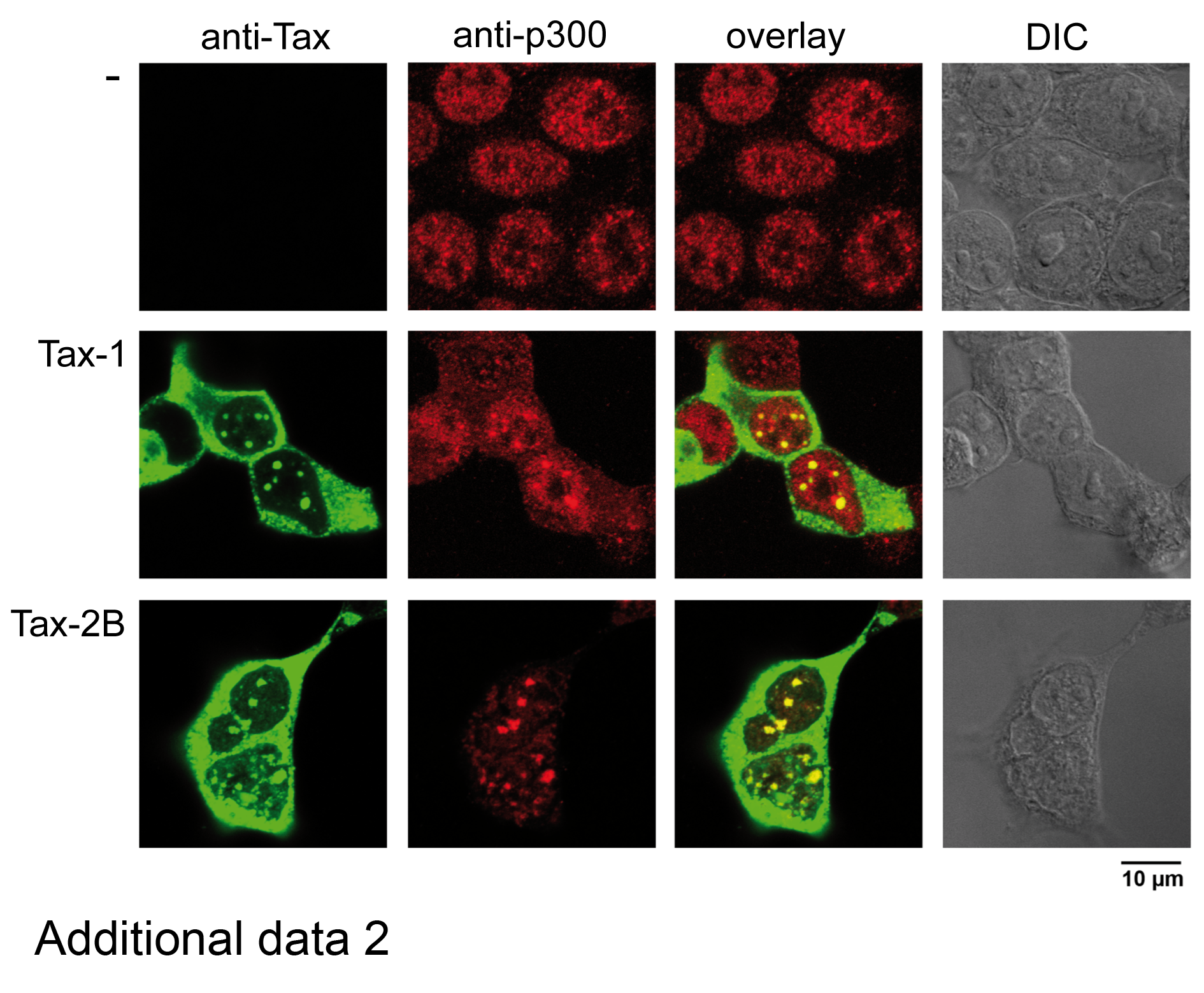

Supplement: Additional file 2 — Tax-1 and Tax-2B colocalize with endogenous p300 in nuclear punctate structures. 293T cells were either transfected or not with the vectors expressing Tax-1 or Tax-2B. The cells were fixed and analyzed by dual immunofluorescence staining with the anti-Tax-1 IgG2a monoclonal antibody or the anti-Tax-2B rabbit polyclonal antibody and an anti-p300 IgG1 monoclonal antibody. The secondary antibodies were goat anti-mouse IgG2a conjugated to Dylight 488, goat anti-mouse IgG1 antibody conjugated to Dylight 649 and goat anti-rabbit IgG antibody conjugated to Dylight 549. The images were collected using a laser scanning confocal microscope. DIC, differential inference contrast. [file 1742-4690-9-102-S2.tiff]

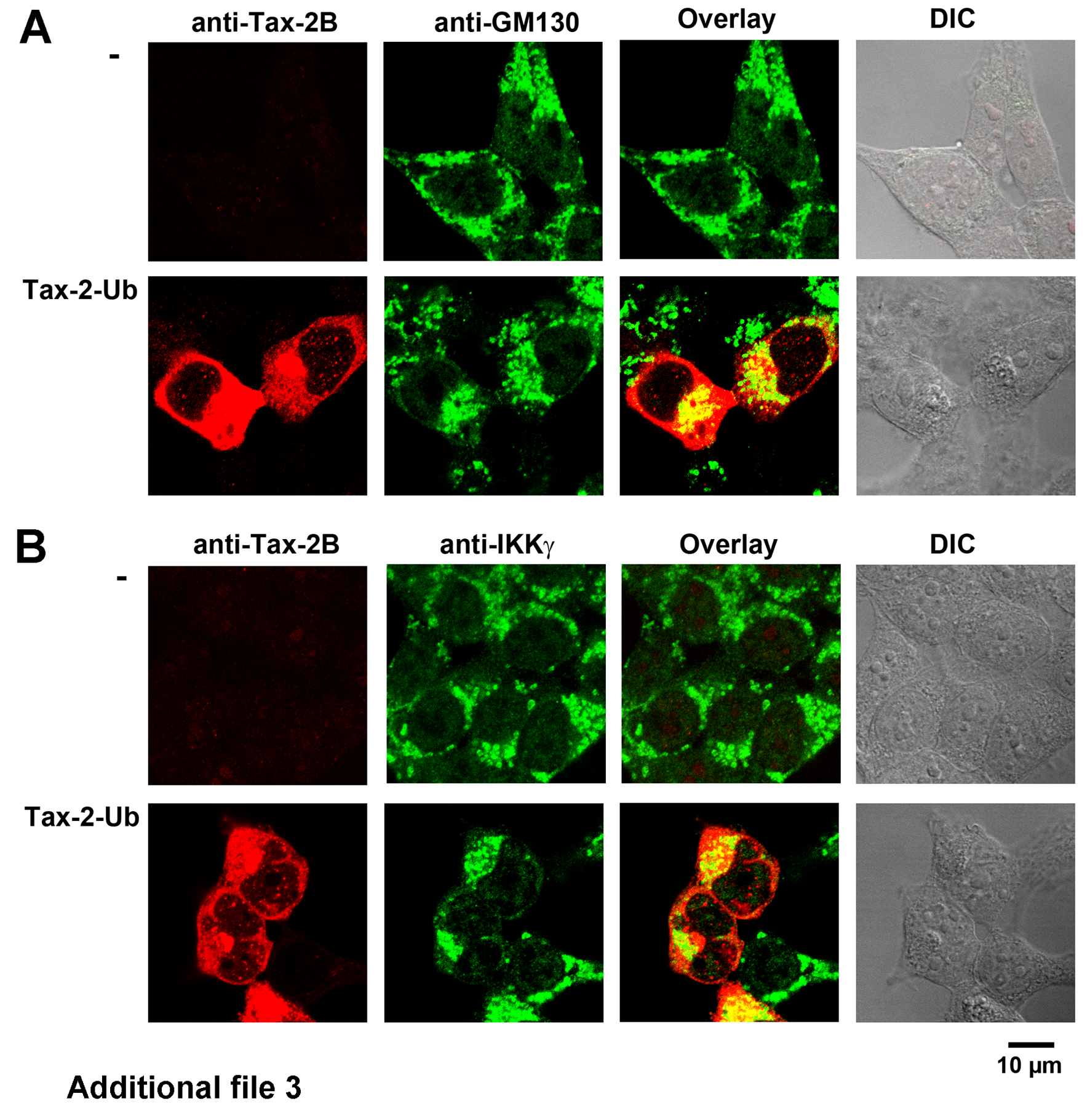

Supplement: Additional file 3 — The wild type Tax-2B fusion to ubiquitin colocalizes with IKKγ in prominent cytoplasmic structures closely associated with the Golgi apparatus. 293T cells transfected or not with the vector expressing the Tax-2-Ub fusion were fixed and analyzed by dual immunofluorescence staining with anti-Tax-2B rabbit polyclonal antibody and (A) anti-GM130 IgG1 mouse monoclonal antibody or (B) anti-IKKγ IgG1 mouse monoclonal antibody. The secondary antibodies were goat anti-mouse IgG1 antibody conjugated to Dylight 649 and goat anti-rabbit IgG antibody conjugated to Dylight 549. The images were collected using a laser scanning confocal microscopy. DIC, differential inference contrast. [file 1742-4690-9-102-S3.tiff]
